# Cosmetic Outcomes and Symmetry Comparison in Patients Undergoing Bilateral Therapeutic Mammoplasty for Breast Cancer

**DOI:** 10.1007/s00268-020-05941-0

**Published:** 2021-02-01

**Authors:** K. Gulis, L. Rydén, P. O. Bendahl, T. Svensjö

**Affiliations:** 1 Department of Clinical Sciences Lund, Division of Surgery, Lund University, Lund, Sweden; 2 Department of Surgery, Kristianstad Central Hospital, 29133 Kristianstad, Sweden; 3 Department of Clinical Sciences Lund, Division of Oncology, Lund University, Lund, Sweden

## Abstract

**Background:**

Breast-reduction techniques are increasingly used in oncoplastic breast surgery. Bilateral therapeutic mammoplasty has the benefit of decreasing breast volume, enabling resection of larger tumors, and the potential to assure good postoperative symmetry. The aims of this study were to objectively asses the cosmetic outcomes of therapeutic mammoplasty in patients with breast cancer, using the breast cancer conservative treatment cosmetic results (BCCT.core) software, to compare this score with the surgeon’s score and the patient’s assessment, and to evaluate if other defined parameters have an impact on cosmetic outcomes. The secondary aim was to compare breast symmetry pre- and postoperatively.

**Materials and Methods:**

We enrolled 146 consecutive patients with primary breast cancer who underwent therapeutic mammoplasty between 2011 and 2018 in Kristianstad Central Hospital, Sweden. We retrospectively collected data from patients’ records. We analyzed the BCCT.core score using postoperative photographs to objectively evaluate cosmetic outcomes on a four-grade scale and compared with preoperative photographs to evaluate symmetry. Cosmetic outcomes were also assessed subjectively by patients and surgeons, using a 10-point Likert scale.

**Results:**

The majority of patients (89%) had good or excellent BCCT.core scores, which correlated with surgeons’ scores, rs =  − 0.22 (*p* < 0.001). Overall, patients were more satisfied with the cosmetic outcomes than the surgeons (*p* < 0.001). Evidence supporting an association between the defined clinicopathological variables, for example, tumor size, and cosmetic outcomes, was weak.

**Conclusion:**

Therapeutic mammoplasty yields a very good cosmetic outcome, evaluated both by subjective and objective measurements. Importantly, symmetry can be improved in patients with asymmetry.

**Supplementary information:**

The online version contains supplementary material available at (10.1007/s00268-020-05941-0).

## Introduction

Breast cancer remains the most common cancer in women worldwide, and cosmetic results following surgery have gained increasing interest in recent decades, both with greater awareness among women and with the introduction of new surgical techniques.

Oncoplastic surgery was introduced more than 20 years ago, with the aim of optimizing both oncological safety and cosmetic outcomes, and studies provide evidence that oncological safety regarding local recurrence and survival are similar to rates after traditional breast-conserving surgery [1–4]. The principles of therapeutic mammoplasty were thoroughly described by McCulley and Macmillan in 2005 [5, 6]; however, subsequent studies involved small cohorts and mainly unilateral cases, very few with bilateral surgery only.

Currently, with 5-year survival rates for breast cancer often > 90% in the developed world, women have greater longevity after they are diagnosed, and having access to an early oncoplastic assessment affects the final cosmetic results. The ideal objective is that every woman diagnosed with breast cancer has the opportunity to obtain the best possible outcome and that each patient’s satisfaction with the results is prioritized. However, the health and economic aspects of oncoplastic surgery must not be disregarded. The need for corrective surgery or re-excision after standard local excision can lead to two or more surgeries instead of one, with recovery times required after each surgery, which adds to the strain on already limited hospital resources. Consequently, there are several benefits regarding oncoplastic surgery for both society and patients.

Therapeutic bilateral mammoplasty is one technique used in oncoplastic surgery, and the technique is a valuable option to consider in patients with breast cancer who also have mammary hyperplasia and good health status [7, 8]. The alternative for these women is mastectomy in patients with larger tumors or multifocal tumors, sometimes with immediate or late reconstruction.

Recent studies have indicated that patient satisfaction with reconstructed breasts is lower compared with satisfaction after partial mastectomies, either following traditional surgery or with an oncoplastic approach [9]. Therefore, the ideal is to maintain the patient’s own tissue, whenever possible. Women with mammary hyperplasia also have a higher risk of toxicity after radiation because of the higher required doses [10].

The objective of this study was to investigate cosmetic outcomes and breast symmetry in patients undergoing bilateral therapeutic mammoplasty for primary breast cancer using the breast cancer conservative treatment cosmetic results BCCT.core software for objective scoring, and comparing the BCCT.core score with the subjective opinion of both surgeons and patients. Our hypothesis was that outcomes following bilateral therapeutic mammoplasty are favorable both objectively and subjectively. The second objective was to investigate changes in symmetry pre- and postoperatively, with the hypothesis that symmetry would improve in patients with preoperative asymmetry. Several predefined variables (body mass index (BMI), multifocality, estimated percentage of breast volume excised, breast volume, extent, ptosis, mammary–jugular distance, axillary clearance, quadrant of the tumor location, smoking, and complications graded according to the Clavien–Dindo classification system [11] were measured to adjust for other relevant factors possibly affecting cosmetic outcomes.

## Material and methods

All patients who underwent bilateral therapeutic mammoplasty with a diagnosis of primary breast cancer between January 2011 and August 2018 at Kristianstad Central Hospital were included in this retrospective observational cohort study.

The surgical procedure was offered to patients with breast cancer who also had marked asymmetry (cancer-affected breast smaller than the other breast), hypertrophy, large tumor extent, or where the location of the cancer would result in unfavorable results with standard wide local excision. The planned approach and related factors for each patient were discussed in both pre- and postoperative multidisciplinary meetings.

**Fig. 1:**
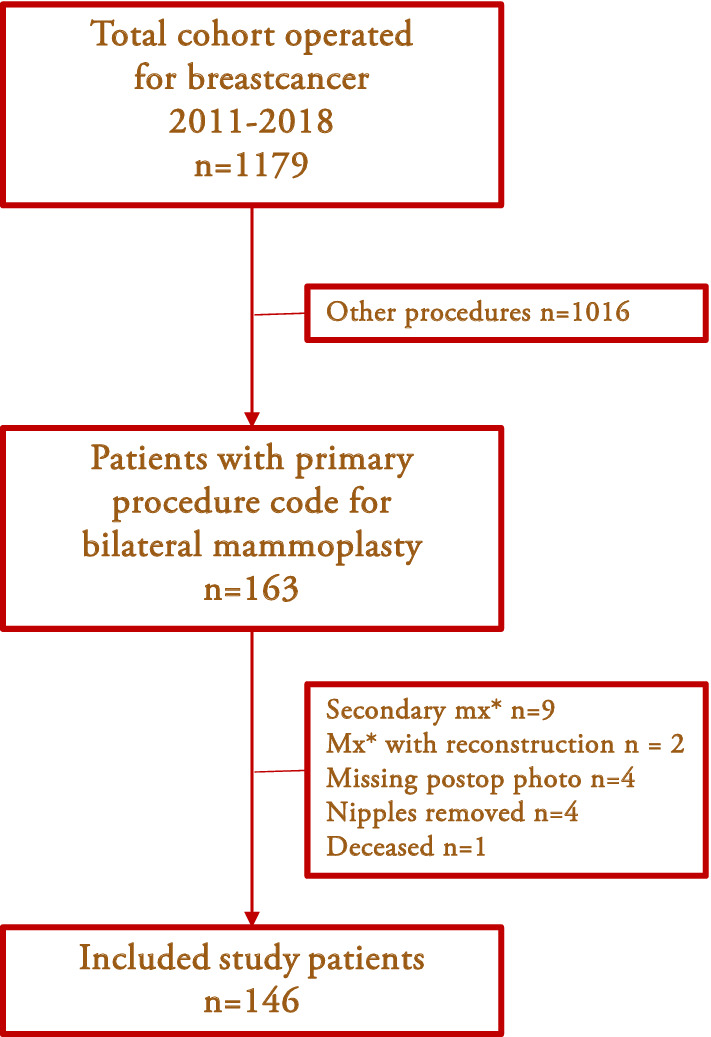
Flowchart of the study *Mx = mastectomy

Exclusion criteria were patients undergoing a secondary mastectomy, missing postoperative photographs, patients whose nipples were removed during surgery, and patients who died before the 1-year follow-up (Fig. 1).

Two patients in the cohort had missing preoperative photographs, and two had missing results from the 10-point Likert scale [12] evaluating the cosmetic outcomes; therefore, results for these patients could not be compared with the results of the BCCT.core scoring.

Clinicopathological data included in the prespecified case report form were collected from our hospital’s patient record system (Melior™; Siemens Healthcare, Upplands Väsby, Sweden) and the operational record system Orbit5™ (Every Healthcare Systems AB, Borås, Sweden). Data extraction was performed by a senior consultant in surgery (TS), and data for at least every tenth patient were independently validated by another surgeon (KG). All information was de-identified, and the information was kept in a coded database.

The clinicopathological data extracted from patients’ records were pre- and postoperative photographs and patients’ age, BMI, breast size, and previous diseases. We also collected data describing the tumor characteristics and patients’ perioperative data, namely mammographic and ultrasonographic size, location of the tumor in the breast, and duration of surgery. The complete list of extracted variables is provided in Supplement 1. Age, tumor size, and positive lymph node status were compared between the included patients and the complete cohort of patients undergoing breast-conserving surgery for breast cancer in our hospital from 2011 to 2018. These data were extracted from the National Swedish Breast Cancer Registry [13].

Before and approximately 1 year after surgery, the surgeon photo-documented patients’ breasts in a frontal projection using an IXUS 95 or 100 camera (Canon Inc., Tokyo Japan). Breast volumes were measured by the surgeon using volume cups at the time of diagnosis [14].

We used postoperative photographs to evaluate cosmetic outcomes and analyzed the preoperative and postoperative photographs to compare pre- and postoperative symmetry; both evaluations were performed using the BCCT.core software [15, 16]. This software evaluates postoperative cosmesis according to specific indices such as asymmetry, color, and scar visibility, to provide an overall cosmetic score, with outcomes graded as 1 (excellent), 2 (good), 3 (fair), or 4 (poor). Symmetry was evaluated quantitatively according to the percentage breast retraction assessment, which is a value calculating the symmetry of the distance between the jugular notch and mamilla in each breast, extracted from the BCCT.core score [17].

Cosmetic outcomes were also evaluated at the 1-year follow-up by all included patients and by the treating physicians. The evaluation was performed using a 10-point Likert scale for cosmetic outcomes, and the specific question asked by the treating physician was “How would you rate the cosmetic outcome on a scale to 1 to 10, where 1 is the worst possible outcome and 10 is the best possible outcome.” The results were registered for patients’ and surgeons’ evaluations, separately.

All patients were recommended to undergo postoperative radiotherapy according to Sweden’s national guidelines. Complications were recorded at the first follow-up visit, approximately 14 days postsurgery, and at the 1-year follow-up. All complications were evaluated by a surgeon.

Complications were recorded for each patient according to the Clavien–Dindo classification system and the presence of complications on the breast cancer-affected side and the contralateral side, separately.

The correlation between the dichotomized BCCT.core scores and patients’ and surgeons’ scores was compared using Spearman’s rank correlation coefficient (r_s_). Significance was analyzed using the Mann–Whitney U test. Wilcoxon’s matched-pairs signed-rank sum test was used to compare surgeons’ and patients’ scores, and also to analyze whether the breast affected with cancer had the same cosmetic outcomes as the contralateral breast.

McNemar’s test was used to analyze whether the breast affected with cancer had the same frequency of postoperative complications as the contralateral breast, and we performed ordinal regression to evaluate the effects of the prespecified variables on the ordinal outcome BCCT.core score. The BCCT.core score was also dichotomized to excellent/good *vs*. fair/poor, and then analyzed with logistic regression.

IBM SPSS Statistics for Windows (IBM Corp., Armonk, NY) was used for the statistical analyses. *P* values should be interpreted as level of evidence against each null hypothesis tested rather than as significant or not according to a cutoff. We made no adjustments for multiple testing. The study was registered in ISRCTN, identification number 82786416, and ethical approval was obtained from the Regional Ethics Review Board at Lund University, Sweden (2018/827). The study had an opt-out option for the patients implemented by an advertisement in the local newspaper in Kristianstad county.

## Results

The final study cohort constituted 146 patients, and the patients’ and tumor demographics are shown in Table 1.

**Table 1:** Patient and tumor demographics

Patient demographics (n = 146)	No	%	Postoperative demographics (n = 151)*	No	%
Age, years			EPBVE^d^		
Median	64		Median	20	
Range	34–90		Range	4.6–67.8	
< 50	22	15	≤ 20	73	53
50–59	31	21	21–40	45	33
60–69	62	42	41–60	16	12
≥ 70	31	21	> 60	3	2
BMI^a^			Missing	9	
Median	28.5		Histological type		
Range	19.9–48.2		IDC	106	70
< 22	9	6	ILC	24	16
22–24.9	21	14	Other types of IC	10	7
25–29.9	59	40	DCIS	9	6
≥ 30	57	39	LCIS and other types of in situ	2	1
Smoking			Histological grade		
Non-smoker	113	81	I	26	19
Ex-smoker	10	7	II	65	49
Current smoker	17	12	III	43	32
Missing	6		Non-invasive	11	
Breast size, ml			Complete remission	6	
Median	1000		Oestrogen^e^ > 10%		
Range	350–2200		Yes	113	82
< 800	46	32	No	25	18
≥ 800	99	68	Unknown	13	
Missing	1		Progesterone^f^ > 10%		
MJ distance, cm			Yes	90	65
Median	29.75		No	48	35
Range	22.5–40		Unknown	13	
Ptosis, cm			HER 2^ g^		
Median	5.5		Yes	16	12
Range	0–14		No	122	88
Indications^b^			Unknown	13	
Asymmetry	40	27	Ki 67 > 30%		
Hypertrophy	79	54	Yes	41	27
Multifocality	25	17	No	110	73
Tumor size	47	32	Unknown	12	
Tumor location	16	11	Lymph node status		
Re-excision	7	5	Benign 7	112	74
Ptosis	24	16	Metastasis	39	26
Quadrant^c^			Tumor size (mm)		
SMQ	42	28	Median	18	
SLQ	71	47	Range	0.5–58	
ILQ	20	13	Extent (mm)^h^		
IMQ	14	9	Median	23	
Central	4	3	Range	3–149	

^a^Body mass index

^b^Multiple indications can be applied for each patient

^c^SMQ:superior medial quadrant, SLQ:superior lateral quadrant, ILQ:inferior lateral quadrant,IMQ:inferior medial quadrant

^d^Estimated percentage breast volume excised

^e^Oestrogen receptor status

^f^Progesterone receptor status

^g^HER2 receptor status

^h^Total size of tumor area, including DCIS

^*^Higher number due to bilateral cancers

The median age was 64 years (range 34–90 years); 39% of the patients were obese (BMI ≥ 30), and 12% were current smokers. The median breast volume was 1000 ml, and the most common indication for surgery was breast hypertrophy. The most prevalent location of the tumor was the superior lateral quadrant. Operative data and additional demographic data appear in Supplement 2.

Compared with all patients who underwent breast-conserving surgery in our hospital during the same years as our cohort, age had good conformity. The larger tumor size in our cohort was expected considering the patient selection criteria (Table 2).

**Table 2:** Demographics for all patients with primary breast cancer, operated in Kristianstad between 2011 and 2018 with breast-conserving surgery, compared to the study cohort

Demographics	Total cohort (n = 1179)	Study cohort (n=146)
Age (median + range)	64 (27–90)	64(34–90)
Tumor size (median + range)	13 mm (0–140)	18 mm (0.5–58)
Positive lymph nodes	22%	26%

The overall rate of complications classified according to the Clavien–Dindo system was 27%, and most complications were grade 1–3. The breast affected with cancer showed a higher frequency of complications (20/146) compared with the contralateral breast (10/146) (Table 3). There was weak evidence for a relevant difference between sides, using McNemar’s test (*p* = 0.10).

**Table 3:** Complications according to Clavien–Dindo Classification, any complications recorded for each breast, types of complications and cosmetic outcome for BCCT.core and Likert scales

	No	%
Clavien–Dindo Classification		
0	107	73
1	12	8.2
2	1	0.7
3	19	13
4	6	4.1
5	1	0.7
Postoperative breast complications*		
No complication in any breast	109	75
Cancer-affected breast only	20	14
Contralateral breast only	10	6.8
Bilateral complication	7	4.8
Types of complications		
Wound defects	13	8.6
Bleeding	11	7.3
Wound infections	6	4.0
Seroma	5	3.3
BCCT.core		
Excellent	40	27
Good	90	62
Fair	16	11
Poor	0	
Likert scale	Median (range)	
Surgeon	8 (3–10)	
Patient	9 (1–10)	

^*^ separately recorded for each breast

**Fig. 2:**
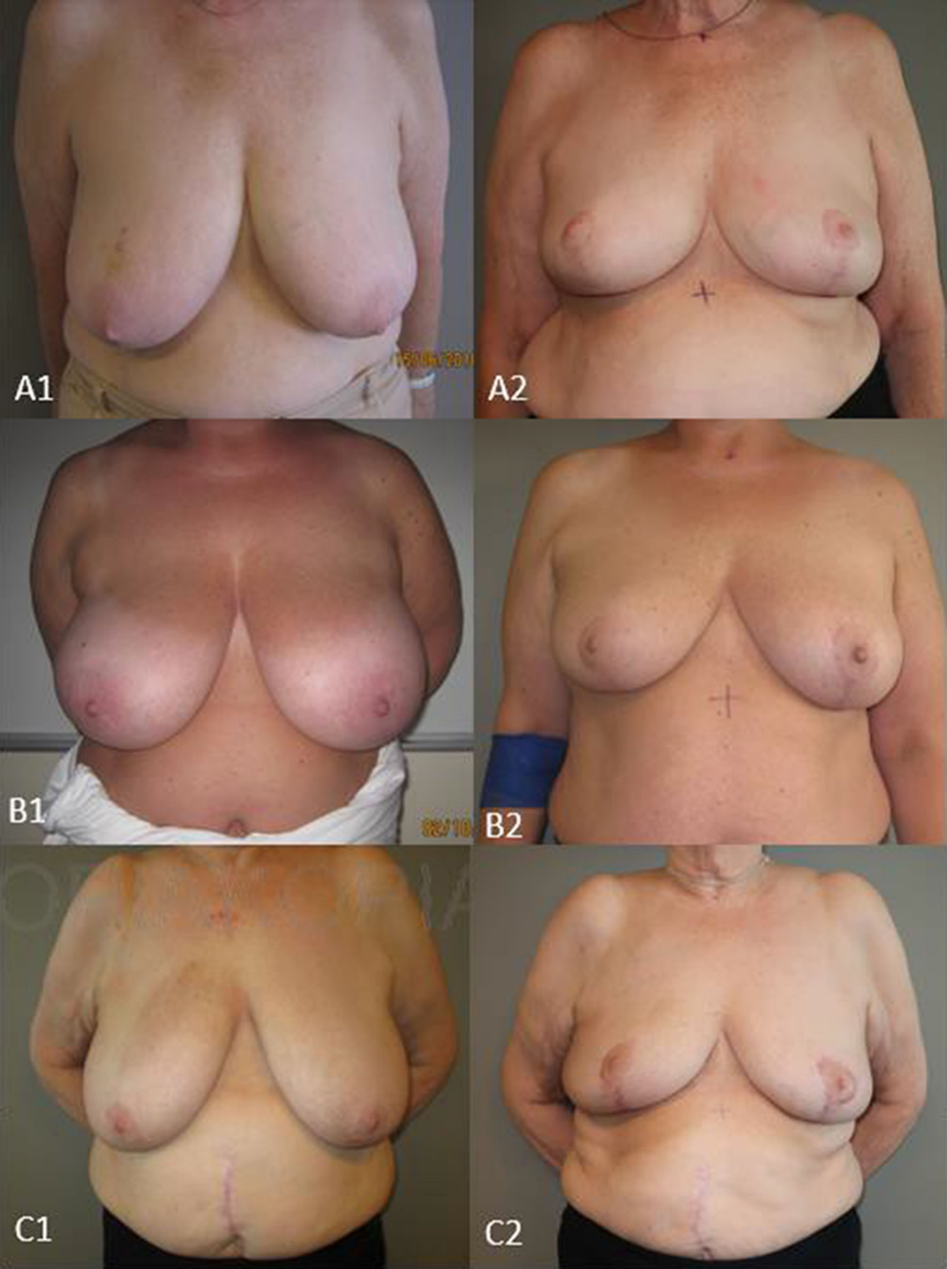
Pre- and postoperative picture by different BCCT.core scores. A2: BCCT.core Excellent”,B2: “BCCT.core “Good” C2: BCCT.core “Fair”

The BCCT.core scores showed that 89% of all assessed patients received a result of good or excellent. None got a result of poor (Fig. 2).

Both surgeons and patients recorded high scores for general cosmetic outcomes, with a median value for the surgeons of 8/10 and 9/10 for the patients; median scores were identical for the cancer-affected breast and the contralateral breast (Table 3).

The correlation between the BCCT.core score and the surgeons’ scores was r_s_ = − 0.23, (*p* < 0.001). The negative value is because the scales in the BCCT.core system and the Likert scale are reversed.

Interestingly, patients were more satisfied with the outcomes than the surgeons (*p* < 0.001). When evaluating the cosmetic results for each breast separately, the Wilcoxon matched-pairs signed-rank test results indicated that patients were more satisfied with the cosmetic outcomes for the contralateral breast (*p* = 0.04). However, the surgeons’ scores showed weak evidence supporting a relevant difference regarding the cancer-affected breast over the contralateral breast (*p* = 0.065).

Overall, symmetry showed no major change postoperatively in either direction, when we included the entire study cohort. However, in a subgroup analysis of five patient’s with ≥ 25% asymmetry preoperatively, there was a positive change in the percentage breast retraction assessment value postoperatively, indicating improved symmetry (Supplement 3).

The prespecified variables showed weak evidence supporting an effect on cosmetic outcomes using the BCCT.core score, in our study, both with ordinal regression and when BCCT.core scores were dichotomized and analyzed with logistic regression (Table 4, Supplement 4).

**Table 4:** Predictors of cosmetic outcome in relation to BCCT.core univariate logistic regression

Logistic regression	OR (95% CI)	*p*
BMI, grouped		
< 22	ref	
22–24.9	0.37 (0.043—3.141)	0.36
25–29.9	0.40 (0.067–2.359)	0.30
> 30	0.41 (0.069–2.453)	0.33
Smoking		
None	ref	
Previous	0.53 (0.064–4.326)	0.55
Active	2.10 (0.400–11.078)	0.38
Volume (ml)	1.00 (0.998–1.001)	0.47
Ptosis (cm)	1.00 (0.832–1.215)	0.96
Mammary–jugular distance (cm)	0.90 (0.771–1.052)	0.18
Axillary clearance*		
Yes	ref	
No	0.44 (0.094–2.029)	0.29
Quadrant		
SMQ	ref	
SLQ	0.32 (0.073–1.432)	0.14
ILQ	3.00 (0.786–11.445)	0.11
IMQ	1.17 (0.200–6.822)	0.86
Central	–	–
Multifocal*		
Yes	ref	
No	0.52 (0.112–2.426)	0.34
Extent (mm)	0,993 (0.967–1.020)	0.60
EPBVE (%)	0.99 (0.953—1.030)	0.64
Clavien–Dindo		
No complication	ref	
Mild (I-II)	1.59 (0.311–8.104)	0.58
Severe 3 <	1.14 (0.294–4.414)	0.96

Contralateral symmetry surgery was made immediately for several reasons. The main one was that the patient will not need a second surgery, and with two surgeons it does not prolong the surgery. Some patients also had mammary hyperplasia and immediate correction is favorable for these patients who will not have to wait for a symmetry correction with different sized breast. Since we have not seen any significant difference in symmetry between the breasts postoperatively, the cosmetic results are satisfactory even though 97% of the patients had radiotherapy to the affected breast.

## Discussion

The strength of this study is its large cohort of patients undergoing pure bilateral surgery (*n* = 146) and the three predefined outcome measures for cosmetic outcome (BCCT.core, and patients´ and surgeons´ assessments). Previous studies evaluating patients undergoing bilateral breast cancer surgery [18–22] involved smaller cohort sizes of 20–82 patients, outcome measures were limited to one or two assessment methods, and only two studies used BCCT.core scores as an objective evaluation model for cosmetic outcomes [20, 21]. In summary, our results confirmed previous findings of very favorable cosmetic outcomes after therapeutic mammoplasty in patients with primary breast cancer, with low surgical complication rates [18–23]. Importantly, we also showed that cosmetic outcomes were not influenced by confounding factors such as tumor size or BMI. Previous studies showed that quality of life improved in healthy women with mammary hypertrophy undergoing breast reduction surgery [23], and a similar effect was shown previously for breast cancer patients [24]. The oncological aspects of breast reduction surgery have been addressed in a meta-analysis [1], and no significant difference was found regarding patients undergoing or not undergoing therapeutic mammoplasty. Additionally, a systematic review showed only positive effects of the oncoplastic approach [8], even after 20 years of follow-up [25].

A retrospective study by Dahlbäck et al. involving a Swedish cohort evaluated postoperative cosmetic results using BCCT.core scores in patients undergoing breast-conserving surgery. However, the study did not compare an oncoplastic surgery group because of the low frequency of this procedure (29/532 patients), in the study [26]. Our cohort had a higher median age compared with Dahlbäck et al.’s cohort (64 years vs 60 years, respectively) and larger tumor size (18 mm vs 15 mm, respectively). Comparing the results in Dahlbäck et al.’s study with our cosmetic outcomes, 15.8% of Dahlbäck et al.’s patients were graded excellent and 57.4% as good, whereas 27.4% of our patients were graded excellent and 61.6% as good. These results indicate the added value of an oncoplastic approach regarding cosmetic outcomes, when evaluated using BCCT.core scores.

The finding that neither tumor size nor the percentage excised breast volume had a relevant impact on cosmetic outcomes is important. These findings indicate that therapeutic mammoplasty is a good option even with large tumors requiring a considerable portion of the breast to be removed.

However, patient selection is an important factor in all oncoplastic surgery, and the indications should therefore be clearly defined to optimize outcome.

The Clavien–Dindo complication rate of 27% in our study is similar to rates reported in other studies using this grading system, with 28.7% in one cohort and as high as 40.5% in a cohort undergoing bilateral mammoplasty [7, 11]. In our study, we studied the complication rates for each side, with a higher incidence of complications in the breast with cancer, and this has not been widely studied before.

One limitation in our study is the retrospective design, even though the surgeons’ and patients’ assessments of the cosmetic results were collected at the same time, at the 1-year follow-up visit. Additionally, the study period spanned 7 years, but the same senior surgeon performing the oncoplastic surgery was part of the surgical team for the entire study period. Furthermore, our results are derived from data from a single hospital in Sweden and therefore must be interpreted with some caution. However, comparing our results with those for the entire cohort of patients undergoing breast-conserving surgery during the same time period, results showed good age and tumor size coherence, which strengthens our study.

Cosmetic results must be satisfying to the surgeon, but also to the patient; therefore, it is important to include patient-reported outcomes, including the patient’s assessment of the cosmetic results, after surgery. Most studies to date are retrospective studies investigating cosmetic outcomes and quality of life, including our study. This means that there is little knowledge regarding patients’ baseline status, which is important when comparing follow-up results.

There is a need for prospective studies comparing baseline and postoperative data to determine how cosmetic outcomes and patients’ quality of life change. Patients with low quality of life at baseline cannot be expected to have a high quality of life postoperatively, but it is important to investigate whether there is a positive effect of oncoplastic surgery compared with standard breast-conserving surgery and mastectomy. We will address this issue in an ongoing prospective trial, NCT04227613.

Bilateral therapeutic mammoplasty yielded very good cosmetic outcomes in patients with primary breast cancer evaluated objectively using a software (BCCT.core) and, importantly, by the patient. Patients were also more satisfied with postoperative cosmetic outcomes than the surgeons. In this study, we found no clinically relevant factors influencing the cosmetic outcome.

## Supplementary information

Below is the link to the electronic supplementary material.

Supplementary file1 (DOCX 20 kb)
