# Supplementary material for: Cosmetic Outcomes and Symmetry Comparison in Patients Undergoing Bilateral Therapeutic Mammoplasty for Breast Cancer
Source: World J Surg. 2021 Feb 1;45(5):1433–41. doi: 10.1007/s00268-020-05941-0 (PMC8026409; doi:10.1007/s00268-020-05941-0)
Supplement: Supplementary file 1 — Supplementary file1 (DOCX 20 kb) [file 268_2020_5941_MOESM1_ESM.docx]

Supplement

1. All variables

| Number | Ptosis right |
| --- | --- |
| Date of surgery | Ptosis left |
| Age | Volume cancer side |
| Side | Volume contralateral side |
| Onesided or bilateral | Bleeding during surgery |
| Quadrant | Time of surgery |
| Indication - assymmetry | ASA |
| Indication - hypertrophy | BMI |
| Indication - multifocal | Smoking status |
| Indication - large tumour | Microscopic size |
| Indication - ptosis | Tumor type |
| Indication - tumour location | Multifocal |
| Indication - re-excision | Histological grade |
| Pedicles | Estrogen receptor status |
| Reduction technique | Progesteron receptor status |
| Secondaty pedicle | Her2 receptor status |
| Cancer in reduction area | Ki67 |
| Rotational pedicle | Axillary status |
| Areola cancer side | Axillary metastasis |
| Areola contralateral side | Marginal |
| Local recurrence | Extent |
| Distant recurrence | Chemoterhapy |
| Fat necrosis | Radiation therapy |
| Regrets surgery | Antihorminal treatment |
| Would do same surgery again | Diabetes |
| Satisfied with surgery | Hypertension |
| Weight of specimen | Asthma/COL |
| Reduction weight cancer side | Heart disease |
| Reduction weight contralateral  side | Rheumatic disease |
| EPBVE | Other lungdiseases |
| Days in hospital | LMWH |
| Re-operation | Acetylsalicyl acid |
| Postoperative bleeding | Surgeons cosmetic score |
| Infection | Patient cosmetic score |
| Seroma | Symmetry score pre-op |
| Complications cancer side | Symmetry score post-op |
| Complications contralateral side | Difference symmetry score |
| Mammographic size | BCCT.core score |
| Ultrasound size | DindoClavien score |
| Mammary jugular distance right |  |
| Mammary jugular distance left |  |

1. Supplementary patient and operative demographics

| **Patient demographics (n=146)** | **No.** | **%** |
| --- | --- | --- |
| **Breast cancer side** | | |
| Left | 67 | 45.9 |
| Right | 74 | 50.7 |
| Bilateral | 5 | 3.4 |
| **Co-morbidity** | | |
| Diabetes | 9 | 6.2 |
| Hypertension | 51 | 34.9 |
| Asthma/COPD | 13 | 8.9 |
| Heart disease | 8 | 5.5 |
| **ASA grade** | | |
| I | 37 | 25.3 |
| II | 93 | 63.7 |
| III | 16 | 11.0 |
| **Radiographic features (n=151)** | **No.** | **%** |
| **Mammographic tumour size (mm)** | | |
| Median | 20 |  |
| Range | 5-80 |  |
| ≤20 (T1) | 84 | 58.3 |
| 21-50 (T2) | 49 | 34.0 |
| >50(T3) | 6 | 4.2 |
| Not visible | 5 | 3.5 |
| Missing | 7 |  |
| **Ultrasonographical tumour size (mm)** | | |
| Median | 17 |  |
| Range | 3-80 |  |
| ≤20 (T1) | 92 | 64.3 |
| 21-50 (T2) | 38 | 26.6 |
| >50(T3) | 1 | 0.7 |
| Not visible | 12 | 8.4 |
| Missing | 8 |  |

| **Operative demographics (n=146)** | **No.** | **%** |
| --- | --- | --- |
| **Operation time** | | |
| Median | 137.5 |  |
| Range | 70-280 |  |
| ≤60 min | 0 |  |
| 61-120 | 49 | 33.6 |
| >120 | 97 | 66.4 |
| **Bleeding** | | |
| Median | 50 |  |
| Range | 0-300 |  |
| ≤50 | 78 | 53.4 |
| 51-100 | 33 | 22.6 |
| 101-200 | 30 | 20.5 |
| >200 | 5 | 3.4 |
| **Hospital days** | | |
| Median | 1 |  |
| Range | 0-15 |  |
| 0 | 13 | 8.9 |
| 1 | 105 | 71.9 |
| 2 | 19 | 13.0 |
| 3 | 5 | 3.4 |
| >3 | 4 | 2.8 |
| **Reduction pattern** | | |
| Inverted T (Wise) | 139 | 95.2 |
| Rackett | 7 | 4.8 |
| **Cancer in reduction area** | | |
| Yes | 64 | 43.8 |
| No | 82 | 56.2 |
| **Pedicles** | | |
| Primary elongated pedicle | 51 | 33.8 |
| Secondary pedicle | 42 | 27.8 |
| **Weight of specimen** | | |
| Median | 166 |  |
| Range | 34-1142 |  |
| ≤50 | 11 | 7.5 |
| 51-100 | 27 | 18.6 |
| 101-300 | 65 | 44.5 |
| >300 | 43 | 29.5 |
| **Reoperation** | | |
| No | 135 | 92.5 |
| Yes, bleeding | 7 | 4.8 |
| Yes, radicality | 1 | 0.7 |
| Yes, axillary clearance | 3 | 2.1 |

1. Symmetry scores

| Patient | Volume  caner side | Volume  contralateral | Side  difference | Pbra pre | Pbra post | Improvement |
| --- | --- | --- | --- | --- | --- | --- |
| 1 | 425 | 600 | 29% | 3% | 2% | 1% |
| 2 | 1000 | 1400 | 29% | 6% | 1% | 5% |
| 3 | 425 | 650 | 35% | 14% | 4% | 10% |
| 4 | 900 | 1200 | 25% | 16% | 6% | 10% |
| 5 | 1100 | 800 | 27% | 17% | 10% | 7% |

1. Ordinal regression

| **Ordinal regression** | (95% CI) | Betakoeff | *p* |
| --- | --- | --- | --- |
| BMI, grouped | (-0.262-0.481) | 0.110 | 0.56 |
| Smoking | (-0.875-0.255) | -0.310 | 0.28 |
| Volume (ml) | (0.000-0.002) | 0.010 | 0.19 |
| Ptos (cm) | (-0.056-0.188) | 0.066 | 0.29 |
| Mammary-jugulary distance (cm) | (-0.109-0.076) | -0.016 | 0.74 |
| Axillary clearance (yes/no) | (-1.463-0.074) | -0.695 | 0.08 |
| Quadrant | (-0.185-0.465) | 0.140 | 0.40 |
| Multifocal (yes/no) | (-1.158-0.558) | -0.30 | 0.49 |
| Extent (mm) | (-0,019-0,010) | -0.005 | 0.53 |
| EPBVE (%) | (-0.023-0.023) | 0 | 0.99 |
| Clavien-Dindo | (-0.262-0.246) | -0.08 | 0.95 |
